# Obstructive Sleep Apnea: From Simple Upper Airway Obstruction to Systemic Inflammation

**DOI:** 10.4103/0256-4947.75770

**Published:** 2011

**Authors:** Ahmad Bahammam

**Affiliations:** From the University Sleep Disorders Center, College of Medicine, King Saud University, Riyadh, Saudi Arabia

Probably, the most important advance in the history of sleep medicine was the discovery of sleep apnea in 1965.^1^ For years, specialists looked into obstructive sleep apnea (OSA) as a simple, intermittent closure of the upper airway; hence, early treatments focused mainly on eliminating airway obstruction. Prior to the 1980s, the only effective treatment for OSA was tracheostomy, which bypasses the upper airway obstruction. The introduction of continuous positive airway pressure (CPAP) therapy through a nasal mask in 1981 marked another important discovery that fueled interest in sleep medicine practice and research.^2^ Since that time, our understanding of the features and consequences of OSA has progressed significantly, and it is now recognized as a major health issue. OSA is a common problem affecting 24% of middle-aged males and 9% of middle-aged females.^3^ In Saudi Arabia, three of ten Saudi middle-aged men and four of ten Saudi middle-aged women are at a high risk of OSA.^4^,^5^ Additionally, 56% of Saudi patients admitted to the coronary care unit with acute coronary syndrome were diagnosed as having OSA with an apnea hypopnea index >10/hour.^6^ The pandemic of obesity has increased the risk of OSA, and the prevalence of obesity is expected to increase. Recent studies have shown that OSA is associated with increased all-cause mortality and risk of cardiovascular morbidity and mortality.^7^,^8^ Additionally, a causal relationship has been established between OSA and some cardiovascular diseases like systemic arterial hypertension. A number of large population studies have shown an association between OSA and the development of systemic hypertension, independent of other confounding factors.^8^,^9^ Evidence also supports an independent association between OSA and ischemic heart disease, arrhythmia, heart failure and stroke.^9^,^10^

The pathogenesis of cardiovascular diseases in OSA patients is not fully understood; however, recent data suggest that the intermittent hypoxia that results from frequent upper airway closure in OSA patients may play a major role in the pathophysiology of cardiovascular complications in OSA.^11^ Intermittent hypoxia is different from the sustained hypoxia seen in patients with chronic lung diseases, and OSA is a unique model for intermittent hypoxia. The short repetitive cycles of hypoxia and re-oxygenation activate different inflammatory processes and release pro-inflammatory cytokines, chemokines and adhesion molecules, which may result in endothelial injury and dysfunction, leading to atherosclerosis.^11^,^12^

In this issue of the *Annals of Saudi Medicine*, Ciftci and colleagues, in a controlled study, explored the effect of CPAP therapy on some of the inflammatory markers that may lead to endothelial dysfunction and atherosclerosis.^13^ They measured vascular endothelial growth factor (VEGF), erythropoietin (EPO), endothelin-1 (ENDO-1) and inducible nitric oxide synthase (iNOS) in OSA patients before and after 12 weeks of CPAP therapy. Initially, serum levels of VEGF and nitrite-nitrate in OSA patients were significantly higher and lower, respectively, compared to controls. Additionally, VEGF levels negatively correlated with nocturnal oxygen saturation. However, treatment with CPAP for 12 weeks significantly reduced VEGF levels and significantly increased nitrite-nitrate levels in OSA patients. No differences in EPO and ENDO-1 were found between OSA patients and controls.

The body responds to severe and sustained hypoxia through a number of adaptive mechanisms that aim to improve tissue perfusion and oxygenation, such as increasing the levels of EPO. Shorter and milder intermittent nocturnal hypoxia may not stimulate the release of EPO or initiate other adaptive mechanisms. Winnicki et al showed an increase in EPO levels in patients with severe OSA; however, EPO levels remained stable in patients with mild OSA.^14^ Therefore, it seems that EPO levels in OSA patients are related to the degree and duration of nocturnal hypoxia, which may explain the discrepancy between different studies. Additionally, VEGF serum levels correlate with the severity of nocturnal hypoxemia. Indeed, Schulz et al demonstrated that patients with severe OSA had markedly increased levels of VEGF compared to patients with moderate OSA.^15^

The favorable and rapid response to CPAP therapy supports the proposed mechanism that it is the intermittent hypoxia that induces the inflammatory changes, rather than a concomitant comorbid condition like obesity, which is prevalent among OSA patients. Long-term longitudinal observational cohort studies have shown a favorable impact of CPAP therapy on cardiovascular outcomes in OSA patients;^9^ however, no long-term randomized trials have assessed the impact of CPAP therapy because of ethical concerns that challenge the execution of such studies. Hence, research addressing the basic molecular mechanisms causing cardiovascular complications in OSA patients and the impact of therapy on those changes may provide novel insights that fill the gap of missing randomized trials. Additionally, such research may open the door for the development of new treatment modalities directed toward suppressing the induced inflammatory cascades in OSA patients. Obviously, large-scale multicenter studies of carefully selected patients and controls, with stringent control for possible confounders, are needed to evaluate the different inflammatory markers associated with OSA, their impact on cardiovascular morbidity and potential treatment modalities.
